# Team-based learning: design, facilitation and participation

**DOI:** 10.1186/s12909-020-02287-y

**Published:** 2020-12-03

**Authors:** Annette Burgess, Christie van Diggele, Chris Roberts, Craig Mellis

**Affiliations:** 1 The University of Sydney, Faculty of Medicine and Health, Sydney Medical School, Education Office, The University of Sydney, Edward Ford Building A27, Sydney, NSW 2006 Australia; 2 The University of Sydney, Faculty of Medicine and Health, Sydney Health Professional Education Research Network, The University of Sydney, Sydney, Australia; 3 The University of Sydney, Faculty of Medicine and Health, The University of Sydney, Sydney, Australia; 4 The University of Sydney, Faculty of Medicine and Health, Sydney Medical School, Central Clinical School, Sydney, Australia

**Keywords:** Team-based learning, Problem based learning, Clinical problem-solving, Medicine and health curriculum

## Abstract

Team-based learning (TBL) provides an active, structured form of small group learning, that can be applied to large classes. Student accountability is achieved through the specific steps of TBL, including pre-class preparation, readiness assurance testing, problem-solving activities, and immediate feedback. Globally, a growing number of healthcare faculties have adopted TBL in a variety of combinations, across diverse settings and content areas. This paper provides a succinct overview of TBL and guidance for teachers towards successful design and implementation of TBL within health professional education. It also offers guidance for students participating in TBL. The paper is informed by both educational theory, and the extensive, seven year experience of the first and last authors in designing, implementing, facilitating and evaluating TBL at a large medical school.

## Background

Team-based learning (TBL) is defined as “*an active learning and small group instructional strategy that provides students with opportunities to apply conceptual knowledge through a sequence of activities that includes individual work, team work, and immediate feedback”* [1]. TBL was originally designed by Professor Larry Michaelsen during the 1980s, in the United States of America, for use in business schools. Michaelsen developed TBL in response to increasing class sizes, and his concern about the effectiveness of learning from lectures to large groups [2]. TBL provided the opportunity to continue teaching in a manner that was engaging, catered for large numbers of students, provided immediate feedback, involved students in decision making, and promoted active small group and class discussions [2]. TBL goes beyond the simple transfer of content, to the application of knowledge through conceptual and procedural problem solving [3]. In recent years, TBL has gained popularity in medical and healthcare education as a resource efficient, student-centred teaching pedagogy, sometimes introduced as an alternative to Problem based learning (PBL). In comparison to PBL, TBL maintains the advantages of small group teaching and learning, but importantly, without the need for large numbers of tutors. Globally, a growing number of healthcare faculties have adopted TBL in a variety of combinations, across diverse settings and content areas [2]. Because of the many variations in the way that TBL is delivered within health professional education, Haidet and colleagues (2012) developed a standardised framework [4]. The aim of this paper is to provide a succinct overview of TBL, and guidance for teachers towards successful design and implementation of TBL within health professional education. It also offers guidance for students participating in TBL. The paper is informed by both educational theory, and the extensive, seven year experience of the first and last authors in designing, implementing, facilitating and evaluating TBL at a large medical school.

## What is team-based learning?

**Fig. 1:**
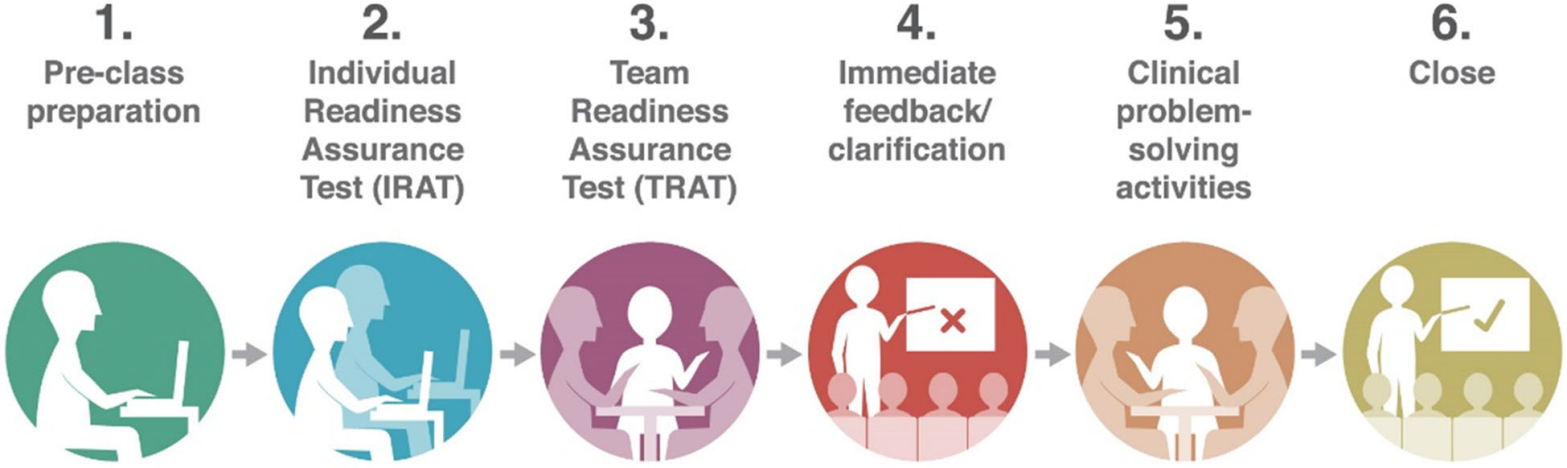
Steps in Team-based learning

TBL provides an innovative approach to student-centred learning, supporting the flipped classroom method of healthcare education [5]. The in-class TBL activities offer an interactive, expert led teaching session that allows a large number of students to work within small teams to apply content to specific problems [6]. The structured format of TBL is illustrated in Fig. 1. This sequenced format provides opportunities to apply and build on conceptual knowledge through a series of steps involving preparation, readiness assurance testing, feedback and the application of knowledge through clinical problem solving activities [2]. An example of this format as applied to TBL at the University of Sydney School of Medicine is provided in Table 1. Through these steps, students are encouraged to self-learn, analyse, communicate, collaborate, speculate, reason, and problem-solve in small teams [2, 6].

**Table 1:**
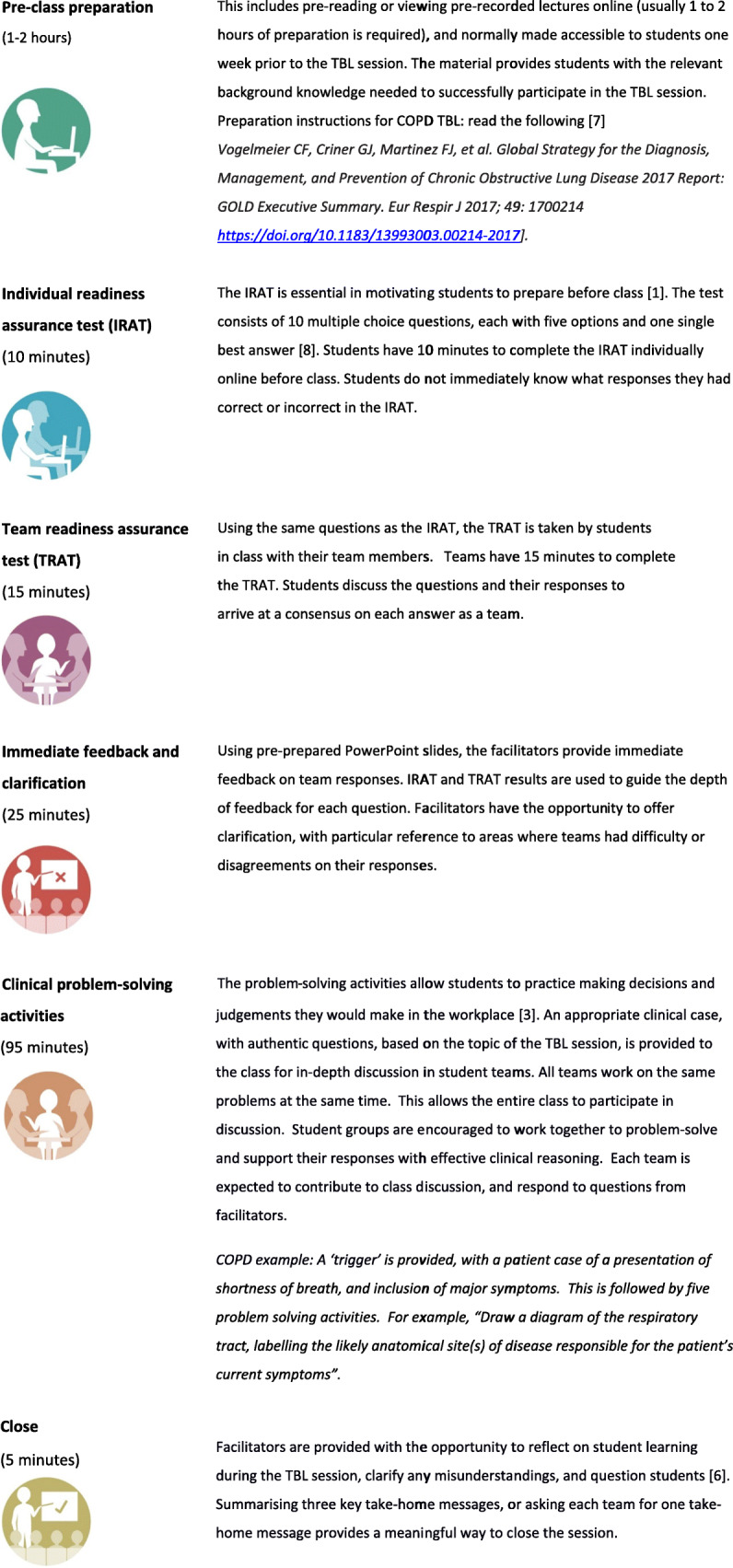
Steps of Team-based learning at Sydney Medical School (each TBL class is 2.5 h), with examples from a patient case based on *Chronic obstructive pulmonary disease (COPD)*

## Why do we use TBL?

*“The primary learning objective in TBL is to go beyond simply covering content and focus on ensuring that students have the opportunity to practice using course concepts to solve problems”* [3]. Research supports the use of TBL, with evidence indicating positive outcomes for students [2, 7–16]. Recent systematic reviews provide evidence of positive outcomes in terms of student experience and academic achievement, particularly when compared to traditional lectures [5, 12, 13]. The interactive nature of TBL encourages healthcare students to develop their communication and collaboration skills, providing a valuable learning experience [10]. The efficient design of TBL addresses resource challenges faced by many higher education institutes. One major benefit of TBL is allowing large numbers of students to experience small group learning, with a small number of expert facilitators [5]. Additionally, students are motivated to complete the pre-reading assigned, as they are held accountable through the readiness assurance testing component of this model [5], resulting in less content being required to be covered during class (‘flipped classroom’). Further, more in class time is allocated to problem solving and critical thinking, promoting greater understanding and retention of knowledge [10].

## Key components of TBL

**Fig. 2:**
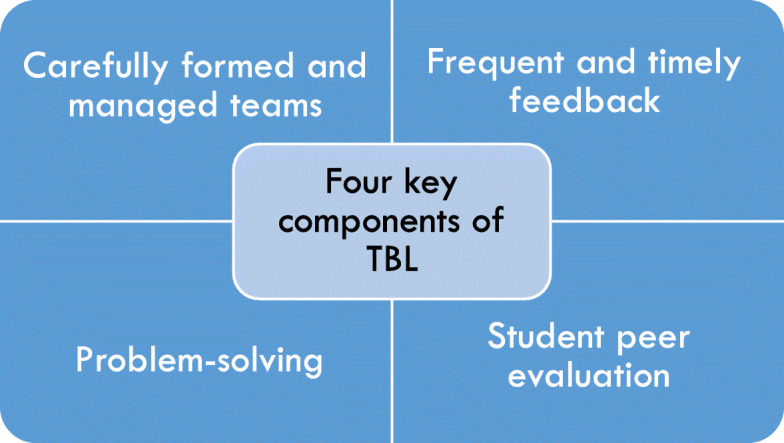
Four key components of TBL

There are four key components of TBL [2, 10, 17], (Fig. 2), including:

### 1) Carefully formed and managed teams

Students should be assigned to teams using a transparent process to ensure there are no pre-existing friendship groups-based teams, and to ensure each team has a diverse mix of students (eg. background knowledge, gender mix, education, training) [17]. Although random allocation methods are likely to prevent self-forming groups of friends, such methods may not adequately achieve the required diversity of learner characteristics within each team [3]. Guidelines recommend that student teams “stay together for as long as possible” [1], to enhance team dynamics, trust and diversity of resources within the group, continuity of learning and cohesiveness of teams.

### 2) Frequent and timely feedback

Feedback is provided to students through the IRAT and TRAT process when answers are discussed immediately after completion of the TRAT, with clarification provided by the facilitators. This immediate feedback is inherent to the TBL process, ensuring that students are provided with an understanding of their level of content knowledge. Facilitators identify gaps in student understanding, challenging students through follow up questions (rather than lecturing), fostering critical thinking. Feedback is key to knowledge acquisition, retention, and influences team development [3, 18].

### 3) Problem-solving

During the clinical problem-solving activities, teams are required to use their collective knowledge, clinical reasoning, ethical views, skills and values to solve complex clinical problems that apply to real life situations [4, 8]. Participation in the problem-solving activities encourages learning and team development through the use of challenging cases. Haidet et al. (2012) recommend that the “four S’s” of problem solving in TBL should always be applied: significant problem, same problem, specific choice, and simultaneous reporting [4]. However, recent literature suggests that the use of “specific choice” (ie Single Best Answer), within health professional TBLs may restrict the potential for students’ discussion and critical thinking [2, 19, 20]. Michaelsen & Richards (2005) have previously highlighted that TBL design within health education may be constrained by a number of predetermined contextual specific elements [17].

### 4) Student peer evaluation

It has been recommended that as part of the peer evaluation process in TBL, students contribute to the grades of other students through provision of quantitative and qualitative feedback to their respective team members [1, 3, 4, 21]. However, it should be noted that peer evaluation may not always provide a meaningful or reliable measure of students’ professional behaviour [22]. Therefore, it may not be suitable as a means of summative assessment, but may provide a useful means of formative feedback to students [22]. Peer evaluation provides an incentive for students to positively contribute to group problem solving and learning, and helps to ensure student accountability [4]. Additionally, giving and receiving constructive feedback is an important professional skill for heath professional students to learn. The practice of giving feedback allows students to develop professional competencies and helps prepare them for their professional lives as clinicians with peer evaluation responsibilities [23, 24]. Receipt of regular, effective feedback has the potential to reinforce good practice, promote self-reflection and insight.

A well developed peer evaluation process, with provision and receipt of peer feedback, is considered key to the success of TBL. TBL literature reports a number of approaches to the “Peer evaluation” method. These methods are generally designed to measure students’ contribution to team cohesion and productivity, as perceived by their teammates, rather than student knowledge [3]. While there are several models for conducting peer evaluation in TBL, students are normally required to provide and receive constructive and professional, written feedback relating to the contributions of team members. One example is “Koles method”, which includes both quantitative and qualitative feedback [25]. The feedback that is provided is rated by the facilitator. Both this score, and the feedback score that is received contributes to the final peer feedback score. The benefit of this method is that the peer evaluation score depends on both the quality of the students’ performance as judged by their peers, and the quality of one’s own feedback. Hence, professional skills in both giving and receiving feedback are enhanced [25].

## Team-teaching in TBL

Traditionally, healthcare curricula have largely been compartmentalized, with teaching delivered in subject based isolation, limiting the opportunity for students to integrate the basic and clinical sciences. However, this integration is foremost in TBL, emphasising the need for team-teaching among disciplines. Team-teaching is described as involving two or more educators working together to cooperatively plan, interact, observe, question and teach, while taking advantage of the special competencies of each educator [26]. Adopting a team-teaching approach to team-based learning provides subject matter expertise in basic sciences, and clinical disciplines. The basic scientists are able to teach and address concepts and questions around the basic science, while the clinicians are able to explain how the basic sciences apply to the clinical case and context. For example, at The University of Sydney Medical School, a TBL teaching team includes one senior clinician, one junior clinician, and one basic scientist, who teach collaboratively and facilitate meaningful learning sessions of theoretical knowledge and clinical application. Teaching is carried out in a unified manner, bringing together different topics to encourage interaction of the basic sciences and clinical disciplines, enabling students to integrate, conceptualise, and apply the newly acquired knowledge. At the same time, both clinicians and basic scientists (senior and junior) build on their knowledge and skills by learning from each other during TBL classes, enriching their own teaching experience.

### Benefits of team-teaching

Benefits for students and facilitators include [27, 28]:

- Students are provided with more than one explanation of complex cases
- Promotes teacher development by peer-teacher observation and reflection on their teaching and learning
- Exposure to different teaching methods and knowledge for both educators and students
- Debate and more active discussions
- Role modelling of interprofessional collaboration
- Brings humour to the classroom

## Feedback to students during TBL

Feedback acts as a continuing part of the instructional process that supports and enhances learning [29]. Feedback is part of an on-going unit of instruction and assessment, rather than a separate educational entity [18]. Feedback promotes learning in three ways [29, 30]:

- Informs the student of their progress
- Informs the student regarding observed learning needs for improvement
- Motivates the student to engage in appropriate learning activities

### Barriers to the feedback process

- Feedback has the greatest impact on students’ knowledge and understanding when it is immediate [31]
- The desire to avoid upsetting students with negative feedback can result in inadequate feedback [31]
- Without external feedback, some students may generate their own feedback. However, self-assessment is often wrong; high performers tend to underestimate their own performance, and lower performers tend to overestimate [32].

## Benefits of TBL for students and facilitators

There are many benefits to TBL for both students and facilitators, and these are summarised in Table 2.

**Table 2:** Summary of the benefits of TBL for students and facilitators

**BENEFITS FOR STUDENTS**	
***Small group experience with facilitators who are experts in their area***	
A clear strength of TBL is having multiple, small groups of students in each room, promoting inter and intra team discussion and peer learning. Having expert facilitators ensures all students are provided with the same, up to date, evidence based guidance and answers [8].	
***Structured learning***	
The specific steps of the TBL process help to engage students. Students move beyond active learning as individuals by participating in structured, collaborative learning activities that are interactive and relevant [32]. Active learning opportunities, which engages participants, will promote deeper understanding and better knowledge retention [33].	
***Students experience the value of working and collaborating in teams***	
Students compare and reflect on their IRAT and TRAT results, and their peers’ contributions to teamwork. Evidence suggests that the worst performing team will usually score higher than the best individual student [3].	
***Students are motivated to reflect on their own strengths and weaknesses as members of a team***	
The peer evaluation prompts students to consider how they can improve as a team member. When implemented correctly, friendly competition promotes student accountability to their ‘teammates’, and to their teachers [7], encouraging students to better prepare for class activities.	
***Students develop professional attributes, such as giving and receiving feedback through peer review***	
Peer review is a common requirement among health professionals, yet it is rarely formally taught and practiced at university [34, 35]. The ability to give feedback is reported to improve communication skills, problem solving, decision making and responsibility [36, 37]. Similarly, receipt of feedback from peers can provide an effective learning experience for students, and create reflective learners, who analyse and reflect on their contributions and performance [38].	
**BENEFITS FOR FACILIATORS**	
**Teaching students who are prepared is more rewarding**	
Staff and students alike value the ‘flipped classroom’ format of TBL. Students are encouraged to prepare for class, and be up to date with course content. Rather than ‘spoon feeding’ content to students, there is time to facilitate meaningful discussion and help students to problem solve [3].	
***Teaching as a team***	
With co-teaching implemented as a strategy in TBL, hospital consultants and university academics come together to develop the students’ knowledge and skills in their areas of expertise. Teaching is carried out in a unified manner, bringing together different topics to encourage interaction of the basic sciences with clinical disciplines, enabling students to integrate, conceptualise and apply this newly acquired knowledge.	
***Facilitators learn from each other***	
Evidence suggests that co-teaching is effective in generating student interest, engagement, knowledge acquisition and retention [39]. At the same time, the teachers may build on their own scientific and medical knowledge, and further hone their teaching skills by learning from each other during TBL classes, ultimately enriching their teaching experience. Our facilitators have described the positive experience of “working with other experts in a collegial atmosphere” as “rewarding” and “positive”.	

## Challenges of design and implementation of TBL

Although there are many benefits to TBL, there are a number of challenges in design, organisation and implementation. The TBL design process is detailed, involving five key steps: 1) identifying learning outcomes, 2) creating problem-solving activities, 3) writing readiness assurance questions, 4) identifying and/or developing preparation materials, and 5) seeking feedback and making improvements [1]. In our own experience, this was a time consuming task, requiring input from a large number of academics, with various expertise [8]. Additionally, faculty development in TBL design and facilitation was needed to ensure academic engagement and understanding of the new teaching method and content, and standardisation in delivery.

Implementation of TBL classes provides some challenges. Although student centred, TBL has an instructional format, with sequenced steps, and completion of all activities within each class requires a disciplined, well organised facilitator [8]. Of the utmost importance is student engagement, understanding and ‘buy-in’ to the TBL process. Students may perceive an increased workload when TBL is introduced [13], particularly because of the flipped classroom format. Students’ motivation to prepare, and contribute to team and class discussion should be encouraged through consistent feedback [20]. Beyond the scope for discussion in this paper, there are a number of resources and software suitable to deliver the various elements of TBL, including pre-readings/videos, IRAT, TRAT, and problem-solving activities. Careful consideration should be given to resource allocation that meets institutional priorities.

## Conclusion

Evidence suggests that TBL provides positive contributions to pedagogy within healthcare education, helping to prepare students for the demands of increasingly complex healthcare systems [1]. Students are attracted to the active, collaborative nature of TBL, and teachers are attracted to the integrated approach of TBL in developing students’ professionalism skills, including leadership, communication and teamwork [40]. As a learning tool, TBL enables a large group of students to participate in small group learning experiences, with a small number of teachers. Although there may be resource saving implications for institutions in terms of required teaching staff, the introduction of TBL may present challenges. Literature recommends that TBL “works best when all of the components are included in the design elements” [41]. Positive aspects of the TBL design include the flipped classroom approach, small groups, testing process, immediate feedback from experts, peer review, and provision of a clinical context by clinicians. The use of a standardised framework, and use of evidence based practice in implementation and facilitation of TBL, will result in better outcomes for students, teachers and institutions.

## Take-home message

**Table:** 

• A growing number of healthcare faculties are adopting TBL, since it allows a large number of students to experience small group learning with experts as facilitators. • Student accountability in TBL is encouraged through pre-class preparation, the IRAT, TRAT, immediate feedback, team problem-solving activities and peer review. • Provision of immediate feedback by experts is essential to student learning throughout the TBL classes. • The challenges of designing and implementing TBL should be considered when allocating resources.	

## Data Availability

Not applicable.

## Abbreviations

TBL: Team-based learning
PBL: Problem based learning
IRAT: Individual readiness assurance test
TRAT: Team readiness assurance test
